# Comparing Ferric Carboxymaltose Versus Iron Sucrose in Patients with Heart Failure

**DOI:** 10.1002/clc.70405

**Published:** 2026-07-07

**Authors:** Kazuhiko Kido

**Affiliations:** ^1^ Department of Clinical Pharmacy West Virginia University School of Pharmacy Morgantown WV USA

**Keywords:** ferric carboxymaltose, heart failure, intravenous iron, iron deficiency, iron sucrose

## Abstract

**Background:**

Limited evidence comparing different intravenous (IV) iron products is available to guide the optimal IV iron product in patients with heart failure (HF) and iron deficiency (IDef).

**Objectives:**

To compare ferric carboxymaltose (FCM) with iron sucrose in patients with HF with reduced or mildly reduced ejection fraction.

**Methods:**

This single‐center retrospective cohort study included patients with HF with reduced or mildly reduced ejection fraction and IDef who received either FCM or iron sucrose. The primary outcome was IDef therapy goal achievement (defined as ferritin 100–300 mcg/L and iron saturation > 20%; ferritin > 300 mcg/L; or hemoglobin > 15 mg/dL regardless of iron study).

**Results:**

A total of 78 patients in the FCM group and 21 in the iron sucrose group were included. The use of FCM was significantly associated with a higher rate of IDef treatment goal achievement after the induction course than iron sucrose (70.1 vs. 41.2%, *p* = 0.026). After adjusting for multiple variables, FCM use was significantly associated with a higher rate of the primary outcome than iron sucrose (adjusted OR 5.8 [95% CI 1.3‐26.1], *p* = 0.022). No significant difference in adherence to the IV iron criteria was found between the two groups (80.8 vs. 81.0%, *p* = 0.985).

**Conclusion:**

Ferric carboxymaltose use was associated with a higher iron repletion rate than iron sucrose in patients with HF with reduced or mildly reduced ejection fraction and IDef.

## Graphical abstract

Limited evidence is available to guide the optimal IV iron product in patients with heart failure and iron deficiency. Ferric carboxymaltose use was associated with a higher iron repletion rate than iron sucrose in patients with HF with reduced or mildly reduced ejection fraction and iron deficiency.

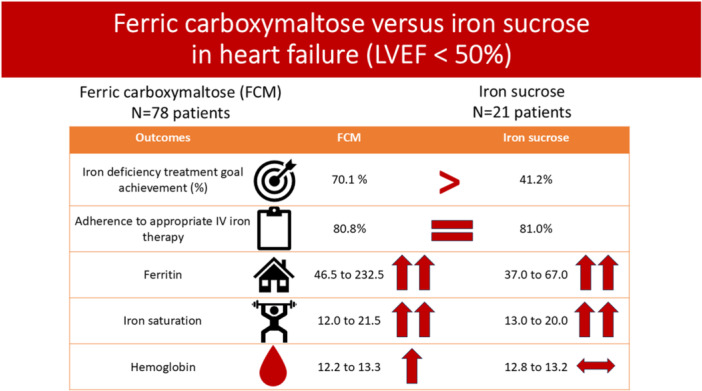

## 1 Background

Iron deficiency (IDef) is prevalent in patients with heart failure (HF). Approximately half of patients with HF experience IDef [1]. Intravenous (IV) iron therapy improves quality of life (QOL) and functionality and potentially reduces hospitalization for HF in patients with HF [1]. The American College of Cardiology (ACC)/American Heart Association (AHA)/Heart Failure Society of America (HFSA) recommends IV iron therapy in patients with HF (LVEF < 45%) and IDef [2]. However, the specific IV iron product is not listed in the guideline, and limited guidance is available for selecting IV iron products because there is limited evidence comparing specific IV iron products in patients with HF. Although the major clinical evidence of IV iron therapy in HF populations used ferric carboxymaltose (FCM), some patients still need to receive other IV iron products (most commonly iron sucrose in the ambulatory care setting based on its clinical evidence in HF [3], its cost, insurance coverage, and availability in infusion centers) [4]. Only one study available comparing FCM with iron sucrose was based on claim data potentially subject to coding errors, and did not have access to the iron study results to evaluate the impact of each IV Iron product on iron study achievement in patients with HF [5].

## 2 Objectives

- To compare FCM with iron sucrose in patients with HF with reduced or mildly reduced ejection fraction based on the rates of IDef treatment goal achievement.
- To provide clinicians with evidence to guide the selection of the appropriate IV iron product for patients with HF and IDef.

## 3 Methods

### 3.1 Study Design

This single‐center retrospective cohort study included patients with HF with reduced or mildly reduced ejection fraction (defined by left ventricular ejection fraction ≤ 50%) and IDef, defined as absolute IDef (ferritin < 100 mcg/L) or functional IDef (ferritin level 100–300 mcg/L and iron saturation < 20%). The patients received either FCM or iron sucrose in an ambulatory care setting between January 1, 2021, and May 30, 2025. All patients were managed by a pharmacist‐led IV iron clinic. Details of the pharmacist‐led IV iron clinic have been discussed elsewhere [6]. Briefly, HF pharmacists collaborate with HF physicians or advanced practice practitioners and manage IV iron therapy under collaborative practice agreements. After the consultation orders were placed, HF pharmacists assessed eligibility for IV iron therapy, developed an IV iron therapy plan, and monitored the course of therapy. Patients received IV iron doses according to the institutional protocols. Iron studies and complete blood counts were monitored 3–6 months after the IV iron induction course. Patients who received IV iron for indications of HF with preserved EF or pulmonary hypertension were excluded from the study. The primary outcome was the IDef treatment goal achievement (defined as ferritin 100‐300 mcg/L and iron saturation > 20%; ferritin > 300 mcg/L; or hemoglobin > 15 mg/dL regardless of iron study). The secondary outcomes included adherence to the appropriate IV iron criteria, follow‐up iron study lab acquisition 3–6 months after the induction course of IV iron, and laboratory values (hemoglobin, ferritin, iron saturation, and phosphorus). The institutional review board exempted this study.

### 3.2 Statistical Analysis

Descriptive analysis used the mean and standard deviation for normally distributed variables and the median and interquartile range (IQR) for non‐normally distributed variables. Categorical variables were presented as counts (%). The Wilcoxon signed‐rank test was performed for two related median values. Pearson's chi‐square test was performed for categorical variables. Multivariable logistic regression analysis was performed to determine the primary outcome. The model included IV iron type (FCM vs. iron sucrose), baseline ferritin, baseline iron saturation, baseline hemoglobin, and body mass index (BMI). A two‐sided significance threshold was used for all statistical tests (*p* < 0.05). No imputation procedures were performed for missing values. All statistical analyses were performed using SPSS Version 29.0.2.

## 4 Results

### 4.1 Baseline Characteristics

A total of 78 patients in the FCM group and 21 in the iron sucrose group were included (Table 1). The mean age was 64.4 ± 14.6 years. The mean LVEF in the entire cohort was 30.5 ± 10.6%. Approximately 83% of IDef was absolute IDef. The median ferritin was 46 [26, 83] mcg/L, and iron saturation was 12.0 [8.0, 16.0]%.

**Table 1:** Baseline characteristics of an iron deficiency cohort.

Characteristics	FCM (*n* = 78)	Iron sucrose (*n* = 21)
Age (year)	65.1 ± 15.0	61.8 ± 13.4
Gender (Male (%))	42 (53.8%)	14 (66.7%)
Race (%)		
White	77 (98.7%)	20 (95.2%)
Hispanic	0	1 (4.8%)
Black	1 (1.3%)	0
SBP (mm Hg)	117.0 ± 17.3	118.4 ± 20.4
HR	76.5 ± 12.3	75.9 ± 13.0
BMI (kg/m^2^)	32.1 ± 9.0	30.1 ± 8.2
Diabetes (%)	45 (57.7%)	12 (57.1%)
Hypertension (%)	72 (92.3%)	21 (100%)
Atrial fibrillation (%)	43 (55.1%)	14 (66.7%)
Previous stroke (%)	16 (20.5%)	6 (28.6%)
History of ASCVD (%)	50 (64.1%)	14 (66.7%)
NYHA‐FC (%)	*N* = 78	*N* = 20
1	6 (7.7%)	0
2	32 (41.0%)	13 (61.9%)
3	38 (48.7%)	7 (33.3%)
4	2 (2.6%)	0
ACC/AHA stages (%)	*N* = 77	*N* = 21
A	0	0
B	1 (1.3%)	0
C	75 (96.2%)	19 (90.5%)
D	1 (1.3%)	2 (9.5%)
Serum creatinine (mg/dL)	1.18 (0.90, 1.48)	1.19 (0.86, 1.30)
Potassium (mEq/L)	4.35 ± 0.47	4.09 ± 0.39
Phosphorus (mg/dL)	3.60 ± 0.63	3.53 ± 0.79
BNP (pg/mL)	279.0 (110.8, 802.8)	156.5 (64.0, 307.0)
Ferritin (mg/dL)	46.5 (27.5, 83.0)	37.0 (26.0, 83.5)
Iron saturation (%)	12.3 ± 6.1	12.7 ± 5.3
Hemoglobin (g/dL)	12.0 ± 2.0	12.1 ± 1.8
Absolute iron deficiency (%)	65 (83.3%)	17 (81.0%)
Iron saturation < 20%	71 (91.0%)	19 (90.5%)
Hb > 14 mg/dL	19 (24.4%)	2 (9.5%)
Left ventricular ejection fraction (%)	30.5 ± 10.5	30.5 ± 11.3
Statin (%)	55 (70.5%)	18 (85.7%)
Beta‐blocker (%)	65 (83.3%, *n* = 74)	20 (95.2%)
ARNI (%)	46 (59.0%, *n* = 74)	15 (71.4%)
MRA (%)	56 (71.8%)	15 (71.4%)
SGLT 2 I (%)	42 (53.8%)	13 (61.9%)

*Note:* Presented as median (interquartile) or *N* (%).

Abbreviations: ACC, American College of Cardiology; ACE‐I, angiotensin converting enzyme inhibitor; AHA, American Heart Association; ARB, angiotensin receptor blocker; ARNI, angiotensin neprilysin inhibitor; ASCVD, atherosclerotic cardiovascular disease; BMI, body mass index; BNP, brain natriuretic peptide; CTEPH, chronic thromboembolic pulmonary hypertension; HFpEF, heart failure with preserved ejection fraction; HFrEF, heart failure with reduced ejection fraction; LVAD, left ventricular assist device; MRA, mineralocorticoid receptor antagonist; NYHA FC, New York Heart Association functional class; PAH, pulmonary arterial hypertension; SGLT 2 I, sodium‐glucose cotransporter 2 inhibitor.

### 4.2 Outcomes

The use of FCM was significantly associated with a higher rate of the primary outcome (IDef treatment goal achievement) after the induction course than iron sucrose (70.1 vs. 41.2%, *p* = 0.026) (Table 2). After adjusting multiple variables (baseline ferritin, iron saturation, hemoglobin, and BMI), FCM was still significantly associated with a higher rate of the primary outcome than iron sucrose (adjusted OR 5.8 [95% CI 1.3–26.1], *p* = 0.022) (Table 3).

**Table 2:** Outcome results of ferric carboxymaltose versus iron sucrose.

	FCM (*n* = 78)	Iron sucrose (*n* = 21)	*p*‐value
Iron study treatment goal achievement, *n* (%) after induction course	47 (70.1%, *n* = 67)	7 (41.2%, *n* = 17)	0.026^#^
Adherence to institutional guidance, *n* (%)	63 (80.8)	17 (81.0)	0.985^#^
Reasons for non‐adherence:	15 (19.2)	4 (19.0)	N/A
No maintenance iron studies in 3–6 months after the induction course	12 (15.4)	4 (19.0)	
Incorrect dose	1 (1.3)		
Inappropriate use	2 (2.6)		
Not all induction doses are given	0 (0)		
IV iron	1250 [750, 1500] mg	1000 [800, 1000] mg	0.007^

^#^Pearson Chi‐Square test.

^Mann–Whitney *U* test.

**Table 3:** Multiple logistic regression analysis for the primary outcome.

Predictor	*p*‐value	Adjusted OR	95% CI (Lower)	95% CI (Upper)
IV iron: Ferric carboxymaltose vs iron sucrose (ref)	0.022	5.809	1.288	26.193
Baseline ferritin (per 1 ng/mL)	0.011	1.026	1.006	1.046
Baseline iron saturation (per 1%)	0.017	1.185	1.031	1.361
Baseline hemoglobin (per 1 g/dL)	0.030	0.700	0.507	0.966
BMI (per 1 kg/m^2^)	0.579	1.018	0.956	1.084

Abbreviations: BMI, body mass index; CI, confidence interval; IV, intravenous.

No significant difference in adherence to the appropriate criteria for IV iron therapy was found between the two groups (80.8 vs. 81.0%, *p* = 0.985). The most common reason for non‐adherence in both groups was the absence of maintenance iron studies in 3–6 months after the induction course (FCM, 15.4 vs. iron sucrose, 19.0%). No significant differences in hospitalization for HF were found between FCM and iron sucrose (11.7 vs. 9.5%, *p* = 0.781).

Table 4 summarizes the impact of each IV iron product on the iron study, hemoglobin, and phosphorus. The follow‐up ferritin and iron saturation levels were significantly higher than baseline values in both groups. However, the increase in ferritin and iron saturation levels at the follow‐ups was numerically higher in the FCM group compared to the iron sucrose group. Follow‐up hemoglobin levels were significantly higher than the baseline value in the FCM group, whereas no significant change was observed in the iron sucrose group. No significant differences in phosphorus levels were observed between the baseline and follow‐up in either group.

**Table 4:** Clinical secondary outcomes between baseline and 3–6 month follow‐up after induction course.

IV iron	Secondary outcomes	Baseline	3–6 months follow‐up	*p*‐value^#^
FCM	Ferritin (mcg/L)	46.5 (27.5, 83.0)	232.5 (135.3, 429.0)	< 0.001
Iron sucrose		37.0 (26.0, 83.5)	67.0 (47.5, 225.0)	0.002
FCM	Iron saturation (%)	12.0 (8.0, 15.0)	21.5(17.0, 27.3)	< 0.001
Iron sucrose		13.0 (7.5, 17.0)	20.0 (18.0, 25.5)	0.002
FCM	Hemoglobin (g/dL)	12.2 (10.8, 13.9)	13.3 (12.0, 14.3)	< 0.001
Iron sucrose		12.8 (11.3, 13.1)	13.2 (12.1, 14.5)	0.068
FCM	Phosphorus (mg/dL)	3.5 (3.2, 3.9)	3.4 (3.2, 4.0)	0.385
Iron sucrose		3.4 (2.8, 3.9)	3.3 (3.0, 3.8)	0.496

*Note:* Presented as median (IQR).

Abbreviations: BNP, B‐type natriuretic peptide; EF, ejection fraction; FCM, ferric carboxymaltose; NYHA, New York Heart Association; WHO, World Health Organization.

^#^Comparison between baseline and 3–6 months follow‐up results (Wilcoxon signed‐rank test).

## 5 Discussion

The present study showed that FCM use was significantly associated with a higher rate of achieving the IV iron therapy goal than iron sucrose. This significant association persisted after adjusting for multiple key variables. In addition, FCM numerically increased ferritin and iron saturation more than iron sucrose. FCM significantly increased hemoglobin levels, whereas iron sucrose did not.

Our study revealed that FCM is more likely to achieve the IDef treatment goal than iron sucrose. However, the total amount of IV iron administered during the induction course was higher in the FCM regimen than in the iron sucrose group (1250 [750, 1500] vs. 1000 [800, 1000] mg) (Table 2). The major clinical trials for FCM used the total amount of FCM from 500 to 2000 mg, depending on hemoglobin levels and weights, while the FERRIC‐HF trial and other non‐HF trials commonly tested the cumulative dose of iron sucrose up to 1000 mg [3, 7, 8, 9, 10]. Thus, the higher IDef achievement rates may be partly attributable to a higher total amount of iron administered rather than to differences in IV iron itself.

The totality of evidence suggests that FCM is the priority agent and iron sucrose is an alternative in ambulatory patients with HF. Although the ACC/AHA/HFSA guidelines for HF do not list the specific IV iron product as a preferred agent, the European Society of Cardiology HF guidelines recommend FCM over other IV iron products [2, 11]. The FAIR‐HF, CONFIRM HF, and AFFIRM‐AHF trials showed the clinical benefits of using FCM in the HF population [8, 9, 10]. Subsequently, the multiple meta‐analyses reassured that FCM was significantly associated with a reduction in hospitalization for HF or mortality compared to placebo in patients with HF [12, 13, 14]. In contrast, one clinical trial, the FERRIC HF trial, showed the benefits of iron sucrose in the HF population [3]. Our real‐world study added to the literature by showing that FCM can replete iron deficits more effectively than iron sucrose, based on a higher rate of achieving the IDef treatment goal.

In addition to the total amount of iron given, the use of FCM may be preferred over iron sucrose based on the frequency of the doses during the induction course (up to 2 weekly doses of FCM vs. up to 5 weekly doses of iron sucrose) and the risk of infusion reaction (FCM has a lower risk than iron sucrose) [4]. Nevertheless, when choosing the appropriate IV iron product, the higher cost of FCM over iron sucrose and the risk of hypophosphatemia may need to be weighed. FCM can cause hypophosphatemia due to increased fibroblast growth factor‐23 levels [15]. However, our research did not reveal a significant change in phosphorus levels at baseline or follow‐up. Similarly, major IV iron trials also showed no significant change in phosphorus levels during follow‐up [15].

### 5.1 Limitations

This is a small‐scale retrospective study, aimed at examining associations rather than establishing causality. Given the limited sample size, the multivariable logistic regression analysis model for the primary outcome incorporated only 4 predictors. This study also lacked sufficient power to detect differences in clinical outcomes, such as hospitalization for HF, between the two groups. This study only included patients with HFrEF or HFmrEF. Thus, these results may not apply to HFpEF, HF with improved EF, left ventricular assist device, or pulmonary hypertension. Although all patients were managed according to institutional protocols, some variations in IV iron dosing were observed across the whole cohort. In addition, the IV iron therapy in the study was primarily managed by pharmacists under collaborative practice agreements with cardiology providers. Thus, the generalizability of the main findings to settings without a pharmacist‐led IV iron clinic remains uncertain. Lastly, this study only included two IV iron formulations of iron sucrose or FCM because these were the most common products in our local practice. Therefore, the findings may not be applicable to clinical scenarios involving other IV iron formulations such as ferric gluconate or ferric derisomaltose.

## 6 Conclusion

Ferric carboxymaltose may achieve iron repletion more effectively than iron sucrose in patients with HF with reduced or mildly reduced ejection fraction. A larger‐scale prospective study is needed to compare ferric carboxymaltose with iron sucrose in patients with HF.

## Author Contributions

The corresponding and primary author performed data collection, analysis, manuscript development, and revision.

## Funding

The author has nothing to report.

## Disclosure

The author used Paperpal to edit and check grammar errors for the manuscript.

## Conflicts of Interest

The author declares no conflicts of interest.

## Data Availability

Data is available on request to the corresponding author.
